# Seroprevalence of Streptococcal Inhibitor of Complement (SIC) suggests association of streptococcal infection with chronic kidney disease

**DOI:** 10.1186/1471-2369-14-101

**Published:** 2013-05-06

**Authors:** Mohan Ganesh Karmarkar, Gouri Pandharinath Hule, Niwrutti Khandu Hase, Preeti Rajeev Mehta, Scott Robert Walter, Kadaba Srinivasa Sriprakash

**Affiliations:** 1 King Edward Memorial Hospital, Mumbai, India; 2 Centre for Health Systems and Safety Research, University of New South Wales, Sydney, Australia; 3 Bacterial Pathogenesis Laboratory, Queensland Institute of Medical Research, Brisbane, Queensland 4006, Australia

**Keywords:** Post streptococcal glomerulonephritis, Chronic kidney disease, End-stage renal disease, Protein creatinine ratio, *Streptococcus pyogenes*, Streptococcal inhibitor of complement

## Abstract

**Background:**

Group A streptococcus (GAS) is an etiological agent for the immune mediated sequela post streptococcal glomerulonephritis (PSGN). In some populations PSGN is recognized as a risk factor for chronic kidney disease (CKD) and end-stage renal disease (ESRD). It was found that a significantly greater proportion of subjects with past history of PSGN than without the history exhibited seroreactions to streptococcal antigens called streptococcal inhibitor of complement (SIC) and to distantly related SIC (DRS). These antigens are expressed by major PSGN-associated GAS types. We therefore predicted that in populations such as India, which is endemic for streptococcal diseases and which has high prevalence of CKD and ESRD, greater proportions of CKD and ESRD patients exhibit seroreaction to SIC and DRS than healthy controls.

**Methods:**

To test this we conducted a SIC and DRS seroprevalence study in subjects from Mumbai area. We recruited 100 CKD, 70 ESRD and 70 healthy individuals.

**Results:**

Nineteen and 35.7% of CKD and ESRD subjects respectively were SIC antibody-positive, whereas only 7% of healthy cohort was seropositive to SIC. Furthermore, significantly greater proportion of the ESRD patients than the CKD patients is seropositive to SIC (p=0.02; odds ratio 2.37). No association was found between the renal diseases and DRS-antibody-positivity.

**Conclusions:**

Past infection with SIC-positive GAS is a risk factor for CKD and ESRD in Mumbai population. Furthermore, SIC seropositivity is predictive of poor prognosis of CKD patients.

## Background

It is estimated that post streptococcal glomerulonephritis (PSGN), an immune mediated sequela of *Streptococcus pyogenes* (group A streptococcus; GAS) infection, afflicts about 472,000 people worldwide contributing to approximately 5000 deaths annually [1]. Because prognosis of PSGN is generally considered excellent, the disease hasn’t received much attention among investigators. However, in the recent decades the knowledge that PSGN is a strong risk factor for chronic kidney disease (CKD) and end stage renal disease (ESRD) in some populations has gained credence [2-5]. A recent prospective study [3] in an Indigenous Australian community found that subjects with history of PSGN were significantly more likely to present with overt albuminurea than the corresponding control subjects (no history of PSGN). Goodfellow et al. [6] found that mean age of onset of proteinuria is significantly lower in patients who are seropositive to streptococcal antigens than in patients who are seronegative, suggesting a role for streptococcal infection in CKD. With alarmingly high prevalence and increasing incidence of CKD and ESRD [7-9], a better understanding of the relationship between these serious diseases and past *S. pyogenes* infection may help to improve management of CKD.

Early epidemiological studies suggested that some GAS M serotypes, notably M1, M12, M49, M55 and M57, are associated with PSGN [10]. Of these, M1 and M57 secrete a protein called streptococcal inhibitor of complement (SIC) [11,12], and M12 and M55 secrete a protein distantly related to SIC (DRS) [13]. In an Australian indigenous population we found significantly greater proportion of subjects with recorded history of PSGN exhibited DRS seropositivity than those without the history [14]. Also, anti-SIC IgM was found to be positively associated with PSGN in Swedish children [15]. Thus, there may be a possible role for SIC, DRS or both in the pathogenesis of PSGN.

As SIC and DRS are highly immunogenic in humans [14,16] and their immune responses are likely to be persistent, serology to these antigens may offer a convenient method to test the hypothesis that seropositivity to SIC or DRS is more prevalent in CKD and ESRD patients than in control subjects. A small comparative study between haemodialysis patients and control subjects from Northern Queensland [17] offers credence to this hypothesis. Furthermore, India with its large population-base, high streptococcal disease burden and high incidence and prevalence of CKD and ESRD [18], provides a unique opportunity to conduct this study. Our results show positive association between SIC seropositivity and chronic renal disease. Furthermore we conclude poor prognosis of SIC-seropositive CKD patients compared to seronegative CKD patients.

## Methods

### GAS strains, study subjects and sera

GAS isolates were recovered from school children to determine circulating types in the community during the study period. GAS strains were typed using the emm typing scheme [19,20]. Approval for swabbing of individuals in the study was granted (EC/Gov/-4/2006) by the Seth G. S. Medical College and KEM Hospital Ethics Committee, India. Written informed consent for swabbing was obtained from the guardians of all children included in the study.

Patients were assessed for renal impairment by a) persistent abnormal serum creatinine levels, b) persistent uremic symptoms (presence of RBCs, pus cells and epithelial cells, proteinurea, increased urine protein:creatinine ratio (PCR)), and c) estimated glomerular filtration rates below 60 ml/min/1.73 m^2^[21] for more than or equal to three months. These clinical parameters were used for diagnosis of CKD and ESRD patients as per KDOQI Guidelines (http://www.kidney.org/professionals/kdoqi/guidelines_commentaries.cfm).

Sera samples from patients and healthy individuals were collected at the KEM hospital and stored at -80°C until used. The study was conducted under ethics approval from Seth G.S. Medical College & KEM Hospital (reference number, EC/GOVT-4/2010). Informed consent was obtained from healthy, CKD and ESRD cohorts.

### Urinary protein creatinine ratio (PCR)

Protein and creatinine in urine samples were measured according to Pesce et al. [22] and Husdan and Rapoport [23] respectively. The PCR for healthy individuals is defined as <0.2 mg protein/1 mg creatinine.

### Measurement of anti-SIC & anti-DRS levels in human sera samples

Serum antibody titers for SIC and DRS was measured as described by Sriprakash et al. [14]. Briefly, high binding flat bottomed immune plates (Himedia) were coated by adding 100 μl of recombinant SIC or DRS [24,25] (100 μg/ml; in carbonate buffer, pH 9.6) per well and incubating overnight at 4°C. After blocking with denatured casein hydrolysate (5% in PBS) for 2 hours, the bound protein was allowed to react with a 1 in 300 dilution of human serum for 1 hour in the same buffer containing 0.05% Tween-20 at room temperature. After washing 5 times with 0.05% Tween-20 in PBS, secondary anti-human IgG antibody conjugated with peroxidase (Sigma-Aldrich) was added and the reactions were detected with 3,3',5,5'-tetramethylbenzidine (Sigma-Aldrich). The reaction was terminated with stopping solution after 30min incubation and optical density (OD) was measured at 450 nm.

### Statistical analysis

Seropositivity based on OD values relies on the use of a cutoff. Due to potential cutoff inflation by the presence of seropositive healthy people, a conservative approach was chosen. This was determined by taking two standard deviations above the mean OD among healthy patients for both SIC and DRS separately then applying those cutoffs to OD values for the CKD and ESRD groups. Differences in characteristics between groups were assessed via tests appropriate to the data. A chi-square test was used for proportions (sex, diabetes) and the Mann–Whitney test was used for skewed variables (age, disease duration). The association between SIC or DRS and each category of renal disease was assessed by calculating odds ratios (OR) for each of the four combinations, and the significance of each association was assessed by Pearson’s chi-square test. Although for some comparisons the expected cell counts in the two by two tables were less than five, p-values from Fisher’s exact test gave similar results to those from Pearson’s chi-square test. We also examined these associations after adjusting for the effects of age and sex through logistic regression. Since none of the controls had diabetes it was not possible to adjust for this comorbidity. However we assessed the effect of diabetes on seropositivity to SIC or DRS among CKD or ESRD patients with logistic regression. Initially univariate models were used to look at the separate (unadjusted) effects of diabetes on SIC or DRS reactivity for each disease group. We then looked at the effect diabetes adjusted for age and sex in a multivariate model.

## Results and discussions

### Study subjects

In all we recruited 100 CKD and 70 ESRD patients attending the clinics at the KEM hospital and 70 healthy subjects. CKD and ESRD status was assessed by perceptible (>1+) precipitation reaction for protein in urine, PCR of >0.2 mg/mg creatinine, positivity for epithelial cells, red blood cells or pus in urine and estimated glomerular filtration rate. The median disease duration as per medical records for CKD and ESRD was 3 years for both with inter-quartile range of 1–4 and 2–5 respectively (p=0.25 by Mann–Whitney test; Table 1). All ESRD patients were undertaking dialysis. Twenty nine percent of CKD and 18.6% of ESRD patients were diagnosed as diabetics while none of the controls had a history of diabetes (Table 1). The diabetes prevalence was marginally higher in CKD than the ESRD patients (chi-square test p=0.12). The median age of both CKD and ESRD patients was 45 years and this was significantly higher than healthy controls (Mann–Whitney test p<0.001 for both). The male:female ratios were 1.7 and 1.6 for CKD and ESRD patients respectively and these did not significantly differ from healthy controls (chi-square test p>0.3 for both).

**Table 1:** Demography and serology of the study population

	**Healthy (n=70)**	**CKD (n=100)**	**ESRD (n=70)**
**Age, median (IQR)**	31 (26–40)	45 (38–55)	45 (36–52)
**Sex ratio, M:F**	2.2	1.7	1.8
**SIC positivity, n (%)**	5 (7.1)	19 (19.0)	25 (35.7)
**DRS positivity, n (%)**	3 (4.3)	7 (7.0)	5 (7.1)
**Diabetes history, n (%)**	0 (0)	29 (29.0)	13 (18.6)
**Disease duration, median yrs (IQR)**	N/A	3 (1–4)	3 (2–5)

All the 70 control subjects (median age 31 and male:female ratio 2.2) showed no more than traces of proteinurea and PCR less than 0.2 mg/mg creatinine). As all the three groups (control, CKD and ESRD) are from Mumbai and surrounding areas, it is assumed that they would have been exposed to a similar range of prevalent GAS types over the years. Of particular interest is to determine whether this population is likely to be exposed to the types capable of producing SIC (*emm*1 and *emm*57) or DRS (*emm*12 and *emm*55).

### Prevalence of SIC or DRS producing GAS types in Mumbai

During the current study period we recovered from Mumbai schoolchildren 51 GAS isolates belonging to 27 *emm* types (Table 2). In our previous study [26] 15 *emm* types were recovered from the same population, and only 3 types are common between these two cross sectional surveys. Taken together, we conclude that GAS isolates in this region are highly divergent as in the rest of the country [27]. However, in these recent two Mumbai-based cross-sectional surveys only a minority of the isolates recovered had the genetic capability to produce SIC or DRS (one isolate of *emm1* and 3 isolates of *emm12*; Table 2). Similar studies conducted on isolates from different parts of India showed that *emm*1 and *emm*57 (SIC-positive types) are in general rarely recovered; and in some studies *emm*12, a DRS-positive type, was recovered [28-33].

**Table 2:** ***emm *type distribution in two cross-sectional surveys**

***emm types***	***Number of isolates***
Current survey (2010–2012)	N=27
63.3; 82.1; st9505; 42.3; 22; 49.4; **12**	3 isolates each
15; 106; 53.9; 73; 86.2; 119.2; st11014; st854; 109.1; 53	2 isolates each
15.1; 60.3; 66; 73.1; 9; st1731.3; stKNB6.0; **1**; 3; 118.5	1 isolate each
Previous survey (2006–2008; [[26]])	N=15
87; 81.2	4 isolates each
25.1	2 isolates each
St9505; 2904; 60.3; 4.4; st211.1; 69; 49; 9; 109.1; 42; 4.5; 44	1 isolate each

SIC and DRS-positive types (*emm1 *and *emm12*) are in bold and underlined.

### SIC and DRS seroprevalence among the healthy subjects from Mumbai area

**Figure 1:**
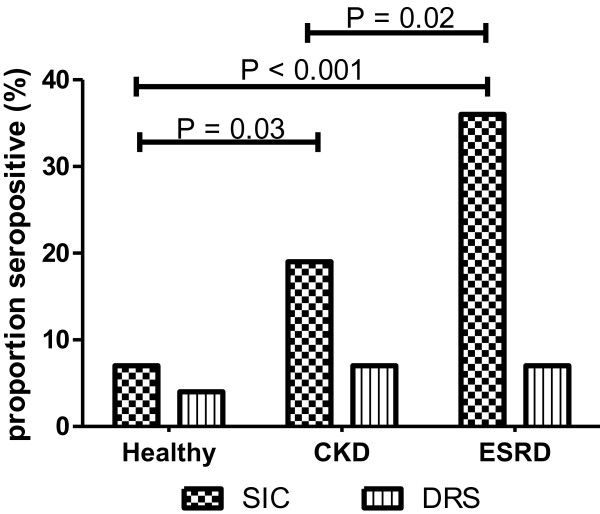
**Proportion of SIC and DRS sero-positive in Healthy, CKD and ESRD cohorts: The cutoff values for SIC and DRS seropositivity are based on the respective titers in the healthy cohort, and were determined as described in the methods section.** P values are only given for the groups (bars) showing significant differences.

Using the cutoff described in the methods section we found that 7.1% and 4.3% of our healthy cohort (n=70) were positive to SIC and DRS antibodies (Figure 1). Despite low isolation rates of SIC- and DRS- strains, high rates of seroconversion to these antigens is not surprising given that they elicit strong and persistent antibody responses. As orthologues of SIC and DRS genes haven’t been found in other organisms thus far, these results suggest that our study population would have been exposed to SIC or DRS producing GAS serotypes sometime in the past.

### SIC and DRS seroprevalence among the CKD and ESRD patients

We further analysed SIC and DRS seroprevalence among CKD and ESRD cohorts. The results revealed that sera from 19% of CKD patients (n=100) and 35.7% of ESRD patients (n=70) reacted with SIC antigen. Thus, relative to the healthy controls significantly high proportion of CKD and ESRD patients are SIC antibody-positive (chi-square p=0.03 and <0.001 respectively) (Figure 1). Antibody positivity to SIC seems to predict increased predisposition for both CKD and ESRD, the OR being 3.05 (95% CI 1.08, 8.61; p=0.04) and 7.22 (95% CI 2.57, 20.28; p<0.001) respectively relative to the healthy group. After adjustment for age and sex the ORs showed a similar although somewhat reduced effect: 2.33 (95% CI 0.75, 7.22; p=0.14) and 3.95 (95% CI 2.16, 21.24; p<0.001) respectively. By contrast, seropositivity to DRS in CKD or ESRD was not significantly different to that in the healthy group whether adjusted for age and sex or not (p>0.3 in all cases).

There was no evidence in this study that SIC seropositivity differed by diabetes status between CKD or ESRD patients in either the unadjusted or adjusted logistic regression models. This suggests that the prevalence of SIC seropositivity is independent of age, sex and diabetes status in the study sample of people with renal disease.

Since stage V CKD patients on dialysis are considered as ESRD patients, we anticipated that the proportion of SIC-positive individuals within each of these cohorts to be similar. However contrary to our expectation, we found that the proportion of SIC-positive ESRD patients is significantly greater than the proportion of SIC-positive CKD patients (p=0.02; odds ratio 2.37 (95% CI 1.18, 4.77)). These results suggest that SIC seropositive CKD patients may progress to ESRD more readily than the SIC seronegative CKD patients.

Lack of correlation between CKD and DRS seropositivity is surprising as the latter is shown to be associated with past history of PSGN in the Australian Indigenous population [14]. It is possible that in Mumbai area DRS-positive S*. pyogenes* (*emm*12 and *emm*55) may not be major types responsible for PSGN. In fact, *S. dysgalactiae* subspecies *equisimilis* (SDSE) is 8 fold more often recovered from the throats of schoolchildren than GAS [26]. SDSE is also known to be an aetiological agent for PSGN [34]. Whereas none of SDSE strains has genetic endowment for SIC expression [35], some strains may possess genes for a DRS-like proteins [36]. However the DRS-like protein from SDSE is immunologically distinct and the antibodies do not cross-react with GAS DRS (our unpublished data). Given these findings, seroprevalence studies against SDSE DRS-like protein is worthwhile, and is the subject of our future study.

## Conclusions

In conclusion we show that past streptococcal infection is an independent risk factor for CKD and ESRD in Mumbai area. Furthermore, our results suggest that SIC serology may have a predictive value for poor prognosis of CKD patients. Early diagnosis of CKD followed by serology to SIC for the Indian population may help a better management of the patients to prevent possible progression to ESRD. A similar study in other endemic regions for these diseases is warranted before this recommendation is adopted into general clinical practice.

## Competing interests

The authors declare no competing interest with this study.

## Authors’ contributions

KSS designed the study and prepared the manuscript; MGK coordinated the study in India. MGK and GPH were responsible of microbiology and serology; PRM facilitated the community and hospital studies; NKH was responsible for nephrology; SRW provided statistical expertise. All authors contributed to intellectual input and helped with the manuscript. All authors read and approved the final manuscript.

## Pre-publication history

The pre-publication history for this paper can be accessed here:

http://www.biomedcentral.com/1471-2369/14/101/prepub
